# Microfluidic Model to Evaluate Astrocyte Activation in Penumbral Region following Ischemic Stroke

**DOI:** 10.3390/cells11152356

**Published:** 2022-07-31

**Authors:** Kathryn M. Denecke, Catherine A. McBain, Brock G. Hermes, Sireesh Kumar Teertam, Mehtab Farooqui, María Virumbrales-Muñoz, Jennifer Panackal, David J. Beebe, Bolanle Famakin, Jose M. Ayuso

**Affiliations:** 1 Department of Pathology and Laboratory Medicine, University of Wisconsin-Madison, Madison, WI 53705, USA; kdenecke@wisc.edu (K.M.D.); bhermes@wisc.edu (B.G.H.); mfarooqui@wisc.edu (M.F.); virumbralesm@wisc.edu (M.V.-M.); djbeebe@wisc.edu (D.J.B.); 2 Department of Neurology, University of Wisconsin-Madison, Madison, WI 53705, USA; teertam@neurology.wisc.edu (S.K.T.); panackal@wisc.edu (J.P.); 3 Department of Dermatology, University of Wisconsin-Madison, Madison, WI 53705, USA; cmcbain@dermatology.wisc.edu; 4 UW Carbone Cancer Center, University of Wisconsin-Madison, Madison, WI 53705, USA

**Keywords:** stroke, astroyctes, microenviroment, microfluidics

## Abstract

Stroke is one of the main causes of death in the US and post-stroke treatment options remain limited. Ischemic stroke is caused by a blood clot that compromises blood supply to the brain, rapidly leading to tissue death at the core of the infarcted area surrounded by a hypoxic and nutrient-starved region known as the penumbra. Recent evidence suggests that astrocytes in the penumbral region play a dual role in stroke response, promoting further neural and tissue damage or improving tissue repair depending on the microenvironment. Thus, astrocyte response in the hypoxic penumbra could promote tissue repair after stroke, salvaging neurons in the affected area and contributing to cognitive recovery. However, the complex microenvironment of ischemic stroke, characterized by gradients of hypoxia and nutrients, poses a unique challenge for traditional in vitro models, which in turn hinders the development of novel therapies. To address this challenge, we have developed a novel, polystyrene-based microfluidic device to model the necrotic and penumbral region induced by an ischemic stroke. We demonstrated that when subjected to hypoxia, and nutrient starvation, astrocytes within the penumbral region generated in the microdevice exhibited long-lasting, significantly altered signaling capacity including calcium signaling impairment.

## 1. Introduction

Stroke is the fifth leading cause of death in the US, affecting 1 in 6 people and causing thousands of deaths and leaving tens of thousands of patients with permanent cognitive damage every year [1,2]. Ischemic stroke originates from brain arterial occlusion and constitutes approximately 85% of strokes [3]. As the brain has little to no oxygen and nutrient reserves, a lack of blood circulation, even for a few minutes, rapidly reduces oxygen and nutrients in the brain tissue, initiating a cascade of events (e.g., cell stress, apoptosis) that rapidly results in cell death and tissue damage [4].

Despite decades of research, treatment options for stroke remain limited with only a few procedures available (e.g., mechanical thrombectomy, or intravenous recombinant tissue plasminogen activator) [5]. With 10% of stroke patients dying, survivors looking at permanent neurological deficits and disability, and 2–4% of all healthcare expenditures are attributed to stroke care, this disease remain a major challenge [6,7]. Furthermore, due to the aging of the world population, and the projected increase in the number of strokes in this population, healthcare expenditure for stroke is projected to increase to almost USD 200,000 M in 2030 [8,9,10]. Overall, stroke imposes a heavy burden on society and there is an urgent need for more efficient therapies to improve cognitive recovery after stroke.

While dead neurons in the anoxic and necrotic core are irretrievably lost, the region surrounding the necrotic core is characterized by hypoxia and low levels of nutrients and contains viable cells. This surrounding area, also known as penumbra, could be potentially salvageable, improving patient cognitive recovery in the long-term [11]. Astrocytes are the most common glial cell and are ubiquitous across the brain, playing a fundamental role in brain homeostasis, cell–cell communication, extracellular environment maintenance, and blood–brain barrier function [12]. Previous studies have shown that activated astrocytes in the penumbral region can play opposite roles after stroke, promoting tissue repair or contributing to further damage depending on the specific molecular pathways involved [13]. Activated astrocytes may exert sustained inflammation by secreting pro-inflammatory cytokines such as TNF-α or IL-1, which in the long-term leads to additional neuronal loss and tissue damage [14]. Conversely, astrocytes can also develop a neuro-protector phenotype that decreases tissue inflammation and promotes oligodendrocyte differentiation, axonal stabilization, and synapse formation [15]. Thus, previous studies tried to stratify astrocytes into A1 or A2 phenotype based on their pro-inflammatory or neuro-protector role [16]. However, this classification proved to be simplistic, since astrocytes seem to exhibit a continuum range of responses after stroke, commonly exhibiting features associated with A1 and A2 phenotype simultaneously. Elucidating the molecular pathways controlling astrocyte response would identify actionable targets to promote tissue repair in the penumbra after stroke.

A barrier to better understanding these pathways is accurately mimicking the oxygen and nutrient starvation during stroke with traditional in vitro and animal models. On one hand, it is challenging to capture the evolving oxygen and nutrient gradients that occur during stroke in traditional in vitro models based on Petri dishes [17]. Organoid models may offer an interesting alternative to generate these gradients, but imaging astrocyte inside of the organoid and selectively retrieving them from the necrotic, penumbral, or normoxic region is challenging. On the other hand, animal models offer a valuable tool, but the animal brain physiology has critical differences compared to the human brain. Additionally, animal models are difficult to monitor on a cellular level and are associated with severe side effects, or even death, posing serious ethical concerns [18].

Microfluidic devices offer an interesting tool to generate complex environments that include oxygen or biochemical gradients [19,20]. Here, we have developed a polystyrene-based microfluidic device to study the effects of the hypoxic and/or nutrient-deprived microenvironment created by an “ischemic stroke” in astrocyte response. Previous studies during the last decades have established PDMS excellent gas permeability, allowing O_2_ and CO^2^ to rapidly diffuse through the microdevice [21]. On the other hand, thermoplastics such as polystyrene present significantly lower permeability [22]. Thus, researchers have leveraged these differences to control oxygen and gas distribution through microfluidic devices. We optimized a polystyrene-to-polystyrene bonding method based on ultrasonic welding to create a sealed chamber, to simultaneously generate gradients of oxygen and nutrients. To decouple oxygen and nutrient gradients we also used a PDMS-based version of our device, which is oxygen permeable and therefore allowed us to generate nutrient gradients while keeping oxygen concentration constant. Our results showed that both hypoxia and nutrient starvation contribute to astrocyte necrosis. Additionally, we observed that astrocytes in the hypoxic region exhibited multiple alterations, including calcium signaling impairment and morphological changes. Finally, we also observed that the hypoxic and nutrient-depleted environment, within the device, led to long-lasting astrocyte impairment that was not reversible even after removing these astrocytes from the microfluidic device, highlighting the long-lasting consequences of the stroke microenvironment on astrocyte physiology.

## 2. Results and Discussion

### 2.1. Microdevice Operation

**Figure 1:**
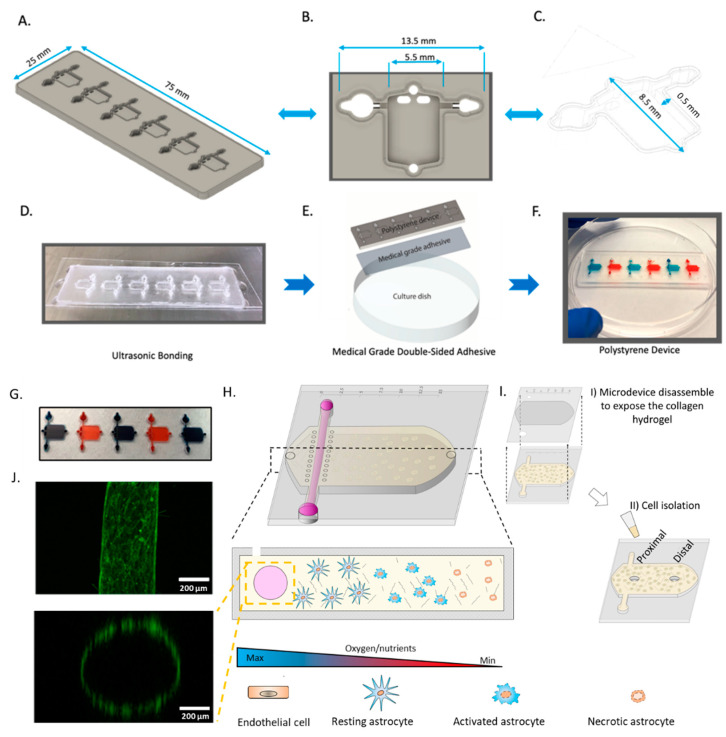
Design and fabrication of polystyrene microfluidic device. (**A**) Side-view schematic of microfluidic device (**B**) Top-down view of the individual microfluidic chamber. (**C**) Side-view of the microfluidic device. (**D**) Picture showing microfluidic device bonded to a 0.175 mm sheet of polystyrene by ultrasonic welding. (**E**) Schematic depicting the use of medical-grade double-sided adhesive bound to 175 mm polystyrene sheet and cell culture flask. (**F**) Image depicting fully assembled polystyrene microfluidic device. (**G**) Picture showing a microdevice array containing 5 culture chambers. The chambers were filled with blue and red die for visualization purposes. (**H**) Scheme depicting the cells in the culture chamber. The asymmetric location of the perfused biomimetic vessel and the diffusion ports create a gradient of oxygen/nutrients across the culture chamber. (**I**) schematic showing the microdevice disassembly for cell isolation. (**J**) Panel illustrating top-view, and cross-section of the biomimetic blood vessel. Microvascular endothelial cells (fluorescent in green) were seeded in the lumen and imaged in a confocal microscope after 24 h in culture.

Ischemic stroke is characterized by a rapid decrease in oxygen and nutrient concentration in the brain tissue caused by a blockage in the brain vasculature. To selectively assess astrocyte response to stroke conditions, we fabricated a microdevice that allowed us to recreate such gradients of oxygen and nutrients. During a stroke, oxygen and nutrients are simultaneously depleted, but the specific role of each factor and how they interact is not completely understood. Therefore, to determine if nutrient starvation alone drives astrocyte death, or both hypoxia and nutrient starvation are needed, we produced two versions of our device: one in polystyrene and one in PDMS. The polystyrene-based device allowed us to simultaneously generate oxygen and nutrient gradients, whereas the PDMS-based device only generated nutrient gradients. The PDMS-based device, which was fabricated using standard SU-8 photolithography, included a tubular structure (hereafter called lumen) lined with endothelial cells to mimic the flow of nutrients in the brain vasculature and a rectangular chamber to seed astrocytes in a 3D collagen hydrogel. Our results demonstrated that the PDMS-microdevice did not generate an observable oxygen gradient after 24 h, whereas the polystyrene-based microdevice showed the formation of an oxygen-gradient after 24 h (Figure S2). Our polystyrene version of the device was fabricated using CNC micro-milling (Figure 1). While the designs are similar, the polystyrene version included a novel feature, consisting of raised edge of polystyrene which allowed us to utilize ultrasonic bonding to adhere a thin sheet of polystyrene to the device (Figure 1B,C). We used ultrasonic welding to generate a stable bonding without the use of a solvent in milliseconds (Figure 1D–G), and no liquid leakage was observed even after 3 days (Figure 1H). To mimic the oxygen and nutrient gradients observed across the infarcted tissue (Figure 1I), the device included the presence of a lumen in one of the flanks of the chamber. We lined this lumen with endothelial cells and perfused culture medium through it to generate a blood vessel surrogate (Figure 1J).

### 2.2. Effect of Oxygen and Nutrient Gradients on Astrocyte Viability

**Figure 2:**
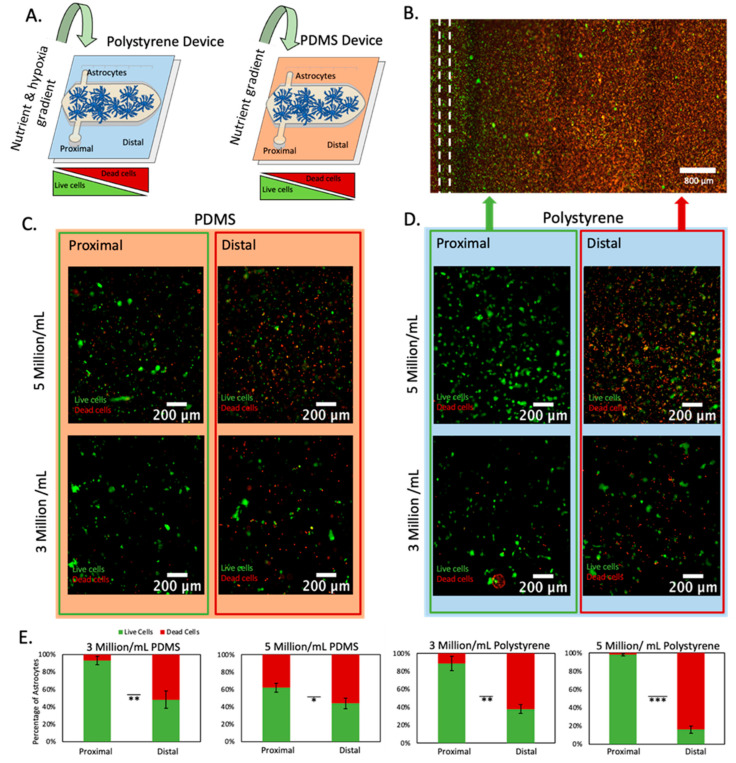
Cell viability analysis at varying densities within polystyrene and PDMS microfluidic devices. (**A**) Schematic representation of polystyrene and PDMS microfluidic devices. Both devices had one lumen located in the culture chamber flank, creating an asymmetric oxygen and nutrient distribution. Since PDMS is permeable to oxygen, only a gradient of nutrient is created in the PDMS-based microdevice. (**B**) Fluorescence microscopy image of astrocytes within polystyrene microfluidic device at 5 million cells/mL. Live and dead cells are shown in green and red, respectively. The location of the lumen is shown in a dashed line. (**C**) Astrocytes within PDMS microfluidic devices at varying densities imaged at lumen and necrotic core. Red and blue backgrounds are used to denote results obtained in polystyrene and PDMS devices respectively. Green and red outlines are used to denote results obtained from the proximal and distal region respectively. (**D**) Astrocytes within polystyrene microfluidic devices at varying densities imaged at lumen and necrotic core. (**E**) Graphs depicting cellular viability of astrocytes at densities of 3 million/mL and 5 Million/mL within polystyrene and PDMS microfluidic devices. *, **, *** denote *p*-value < 0.05, 0.01, 0.005 respectively.

Next, we set out to study astrocyte response to nutrient and/or oxygen starvation. We stained astrocytes with anti-GFAP to confirm their authenticity (Figure S1). We decided to study to what extent nutrient starvation alone could contribute to astrocyte death in the infarcted area. Thus, we fabricated two microfluidic devices, one of them based on PDMS, which is permeable to oxygen and maintains the cells oxygenated; and the second one based on polystyrene, which is impermeable to gas, allowing us to generate gradients of nutrients and oxygen simultaneously (Figure 2A). We embedded astrocytes in a 3D collagen hydrogel and then perfused media and cultured the cells within the device for 3 days to generate a gradient of necrosis (Figure 2B). After three days in the device, we performed a live-dead staining with we disassembled the device to expose the collagen gel with the astrocytes embedded and stained viable and dead cells with CAM (green) and PI (red), respectively. In both the PDMS device and the polystyrene microdevice, we observed cellular viability decreased as we increased the distance from the perfused vessel (Figure 2C–E). Additionally, we observed cell death increased in both the PDMS device and polystyrene device as we increased the cell density from 3 million/mL to 5 million/mL, which is compatible with a higher nutrient demand due to the presence of more cells in the microfluidic device (Figure 2E). More importantly, the presence of necrosis in the PDMS-based microfluidic device demonstrated that nutrient starvation alone generated astrocyte necrosis, whereas combined nutrient and oxygen deprivation significantly increased astrocyte necrosis. 

### 2.3. Nutrient and Oxygen Starvation Leads to Long-Lasting Changes in Astrocyte Calcium Signaling

**Figure 3:**
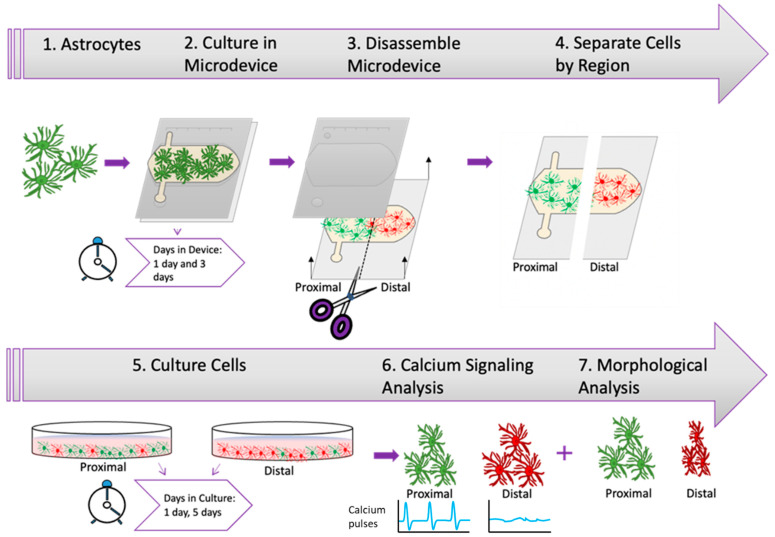
Experimental Design. Illustration depicting experimental timeline beginning with the harvesting of rat astrocytes and culture within microfluidic device at different time periods followed by the extraction of astrocytes from device and culture of astrocytes prior to calcium signaling analysis and morphological analysis.

As described before, astrocytes affected by oxygen and nutrient starvation can exhibit sustained pro-inflammatory or neuroprotector responses. In this context, one of the damaged-associated signaling mechanisms used by astrocytes are calcium waves or pulses, which travel across the brain faster than other chemokines due to the small size of calcium. Thus, we decided to analyze whether astrocyte calcium signaling was affected during and after the stroke-induced nutrient/oxygen starvation. Our previous results showed that necrosis only appeared after 3 days in the device. Therefore, we cultured astrocytes in the device for 1 or 3 days and then isolated them from the proximal and distal region and cultured them again in regular flasks to study whether they exhibit long-lasting impairment in their calcium signaling pattern (Figure 3). 

**Figure 4:**
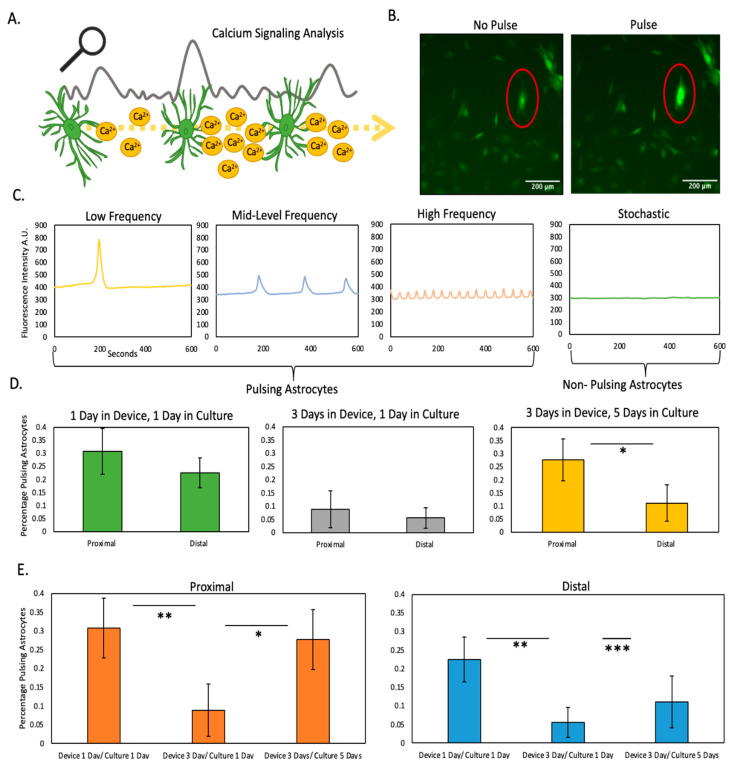
Calcium signaling analysis. (**A**) Illustration depicting analysis of calcium waves generated by astrocyte signaling (**B**) Image depicting astrocytes stained with the calcium-sensing dye. Red outline highlights a pulsing astrocyte (**C**) Graph illustrating differing types of astrocyte calcium pulsing (**D**) Graphs analyzing percentage of pulsing astrocytes from device illustrating differences at proximal and distal locations at varying time points (**E**) Graphs analyzing percentage of pulsing astrocytes from device illustrating recovery potential of astrocytes at proximal and distal locations. *, **, *** denote *p*-value < 0.05, 0.01, 0.001 respectively.

After isolation from the microfluidic device, astrocytes were reseeded in traditional culture flasks for a period of 1 or 5 days prior to analyzing calcium pulsing (Figure 4A–C). The results demonstrated that after 1 day in the microfluidic device, followed by 1 day in traditional flasks, astrocytes from the proximal and distal regions had similar calcium pulsing pattern. Interestingly, after 3 days in the microfluidic device and one day in culture, astrocytes from both the proximal and distal region exhibited a lower level of calcium pulsing compared with only 1 day in the microfluidic device, yet proximal and distal were similar to each other (Figure 4D). This observation is compatible with the viability cell analysis (Figure 2), which demonstrated that 3 days were required to induce necrosis in these cells. Interestingly, after 3 days in the microfluidic device and 5 days in traditional culture, the astrocytes from the proximal region recovered the pulsing pattern observed before the necrosis happened (i.e., 1 day in the device) (Figure 4D green graph vs. yellow graph). Interestingly, astrocytes from the distal region did not show any recovery after 4 days in the flasks, and exhibited persistent disruption in their calcium pulsing pattern (Figure 4E). These results suggest that astrocytes located in the proximal region recovered from the stress experienced in the microfluidic device, whereas viable astrocytes located in the distal region suffered long-lasting calcium signaling impairment, which in turn could explain the sustained activation observed in the hypoxic penumbra observed in animal models Figure S3. 

### 2.4. Morphological Analysis

**Figure 5:**
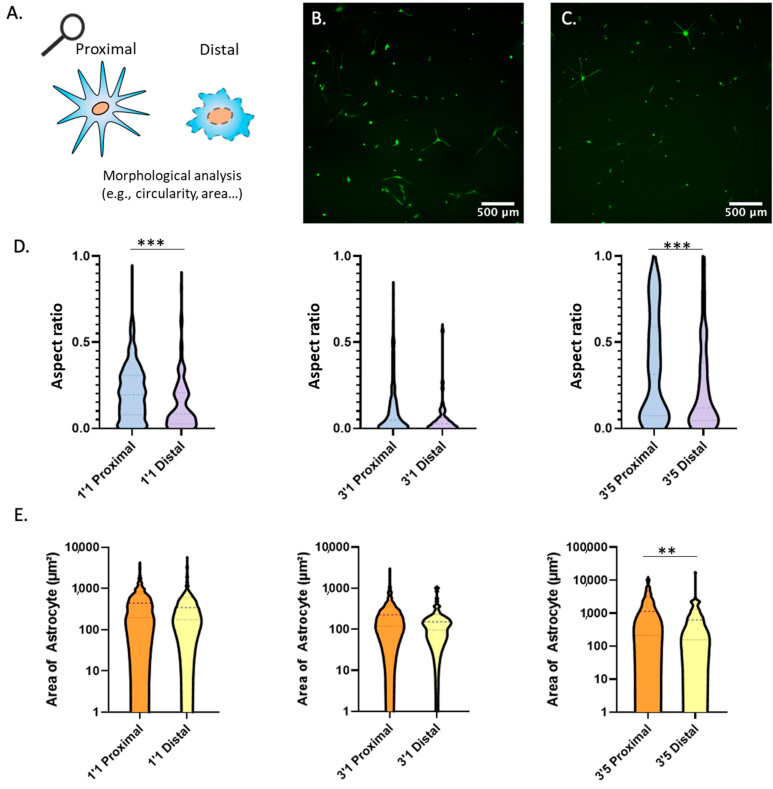
Morphological Analysis. (**A**) Illustration depicting methodology for circularity morphological analysis. (**B**) Astrocytes extracted from the proximal region of the device after 3 days and placed in culture for 5 days prior to imaging. (**C**) Astrocytes extracted from the distal region of the device after 3 days and placed in culture for 5 days prior to imaging. (**D**) Violin graphs analyzing circularity of astrocytes at multiple time points after extraction from proximal and distal regions of the device. (**E**) Violin graphs analyzing area of astrocytes at multiple time points after extraction from proximal and distal regions of the device. ** and *** denote *p*-value < 0.01 and 0.005 respectively.

Astrocytes commonly exhibit numerous cellular projections that they use to establish contact with other cells such other astrocytes, neurons, or blood vessels (e.g., neurovascular units). Thus, we decided to investigate whether nutrient and oxygen starvation had long-lasting effects of the capacity of astrocytes to generate these cellular projections. We embedded the astrocytes within the collagen gel at a density of 5 million cells/mL within the polystyrene device. Next, we cultured the astrocytes in the following conditions: one day in the device and one day in culture (D1/C1), three days in the device and one day in culture (D3/C1) and three days in the device and five days in culture (D3/C5) (similar conditions to Figure 5). We analyzed the aspect ratio of astrocytes extracted from the proximal and distal region (Figure 5A). Thus, astrocytes forming numerous cell projections have a high aspect ratio, whereas rounded and circular astrocytes have a lower aspect ratio. We found a statistically significant difference in the circularity of the astrocytes between proximal and distal regions at time points D1/C1 and D3/C5 (Figure 5B–D). Thus, similar to calcium pulsing, astrocytes located in the distal region showed significantly different morphology compared with those ones located in the proximal region after 5 days post retrieval (D3/C5). Further, we analyzed the cellular area of the astrocytes extracted from the proximal and distal region and found a statistically significant difference at time (D3/C5) (Figure 5E) (Figure S4 for higher magnification images). From these results, we concluded that after a period of oxygen and nutrient starvation in the device, astrocytes from the distal region showed long-lasting differences in cell morphology compared with astrocytes from the proximal region. 

## 3. Conclusions

Despite the devastating global burden posed by stroke and decades of investment in research, there remains a dire need for neuroprotective and regeneration-promoting strategies. Due to their prominent role during all phases of stroke, and the biphasic protective and deleterious effects, astrocytes are a promising therapeutic target to improve cognitive recovery after stroke^21^]. Our findings offer an interesting platform harnessing novel in vitro technology to elucidate functional and morphological changes in astrocytes affected by stroke-induced hypoxia and nutrient starvation. Additionally, these platforms could be used to continue exploring the effects of nutrient, oxygen, or nutrient and oxygen starvation in astrocyte biology [22]. Although in this study we performed a semi-quantitative analysis of the oxygen profile, previous studies have demonstrated the potential of microfluidic devices for sensor integration [23]. Thus, oxygen sensors could be integrated in microfluidic devices to potentially provide real-time monitoring of the oxygen profile. As new technologies develop, advances in mass spectrometry imaging (MSI), Raman infra-red microscopy, or FTIR could provide versatile tools to monitor nutrition and metabolism starvation in real-time [23] Current MSI technologies commonly require aggressive sample preparation that limit their application for 3D samples that include live cells. On the other hand, Raman and FTIR microscopy imaging systems offer a non-destructive alternative to monitor metabolite evolution in real-time, although they still require complex expertise and metabolite spectra deconvolution remains challenging. Overall, additional technological improvements are still necessary, but these technologies may offer a promising approach to monitor nutrient and metabolite consumption in complex biological samples such as microfluidic systems.

In particular, we observed decreased calcium signaling both in proximal and distal astrocytes showed limited recovery potential depending on the level of stress experienced (i.e., astrocytes in the distal region did not recover the initial pulsing pattern). These findings were particularly interesting as astrocytic calcium signaling is well established as undergoing spatiotemporal alterations following ischemic damage [23]. The fluorophore used in this study to detect calcium pulses (fluo-4 AM) is activated by esterases once it is inside of the cell, increasing its fluorescence and making it cell impermeable to prevent leakage to the extracellular medium. Thus, in this study we limited our analysis to intracellular pulses. However, future studies could explore alternative formulations to study the effects of oxygen and nutrient starvation on astrocyte calcium secretion. Future approaches aimed at modulating calcium signaling in reactive astrocytes may elucidate these signaling changes and potentially ameliorate stroke-induced pathophysiology. Our experiments demonstrated the capacity to retrieve live cells from the device, allowing researcher to recessed them to evaluate long-lasting effects on the cellular function such as calcium signaling. However, microfluidic devices are also compatible with traditional molecular biology techniques such as RT-qPCR, ELISA, or luminex assays.

Furthermore, we determined that impaired functional recovery was more pronounced in the oxygen and nutrient starved distal astrocytes. These findings imply that once oxygen and nutrient levels are restored, pathological changes persist and potentially give rise to stroke-induced epigenetic modifications [24]. Such changes in gene expression may have major implications for cell plasticity in the brain, including the capacity to repair tissue damage and restore lost functionality and subsequent recovery [25]. As previously noted, astrocytes become reactive in response to cerebral ischemia and undergo functional and morphological changes which elicit both beneficial and detrimental effects [14,26]. Our technology could permit an in-depth focus upon such alterations, along with precipitating factors, and the potential for astrocytes to return to a naive state. In addition to the damage sustained during cerebral ischemia, subsequent reperfusion can potentially contribute to injury [27,28]. We envision that our platform could be leveraged to explore these mechanisms contributing towards ischemic reperfusion-injury, including inflammatory responses and oxidative stress, along with potential therapeutic interventions.

A potential limitation of the study was the exclusive focus upon astrocytes. Although astrocytes are crucial players in the stroke, other cells, including neurons, microglia, oligodendrocytes, endothelial cells, and pericytes are also altered by the ischemic cascade [29,30]. Consequently, there is scope in future studies to incorporate multiple cell types such as microglia, pericytes, and neurons to comprehensively assess the temporal effects of oxygen and nutrient deprivation on morphology and functionality. Similarly, we limited our study to collagen type 1, but other matrix formulations could be explored in the microdevice. In this context, previous studies have shown that both matrix biochemical composition and mechanical properties such as stiffness can have profound effects on astrocyte biology [31]. Overall, microfluidic platforms offer a versatile platform to decipher the role of specific biochemical and cellular factors in a more holistic manner [32].

The use of rat, rather than human, astrocytes is an additional aspect that should be addressed by future studies. Rodent astrocytes exhibit fundamental differences to those found in the human brain; human astrocytes display greater heterogeneity, and are more complex with many more cellular projections than those observed in the rat [33]. Interspecies differences notwithstanding, rodent astrocytes have typically been preferred in research as the harvesting of human brain cells has been beleaguered by both technical and ethical considerations. However, the use of induced human pluripotent stem cells may circumvent the limitations of cells extracted directly from humans and their application, in conjunction with microfluidic platforms, offer exciting opportunities to further bridge translational gaps between animal models, traditional in vitro platforms, and clinical applications.

## 4. Materials and Methods

### 4.1. Polystyrene Fabrication

CNC micro-milling was used to fabricate the top layer of the device. The devices were designed using Fusion 360 and contained six chambers, which were milled out of 2 mm polystyrene. To minimize gradients in the Z-direction, the culture chamber was only 500 μm-high with a 250 μm-high ceiling. The chamber was 5 mm-width and 10 mm-long The device was removed from the mill and cleaned using an air gun and isopropyl alcohol (IPA). Polystyrene sheets of 0.175 mm thickness were cut to the dimensions of the device using a laser cutter. Ultrasonic bonding was used to affix a 0.175 mm polystyrene sheet to the top of the device. Double sided, medical-grade adhesive tape (Adhesives Research, Cat: 90106, Lot: 388661) was affixed to the device on top of the. 175 mm polystyrene sheet. The device was then adhered to a cell culture dish (Corning, Cat: 353003, Lot: 6318018). Prior to use in cell culture, the devices were sterilized with a 15 min exposure to ultraviolet light and individual chambers were treated with ethanol followed by DI water. 

### 4.2. PDMS Fabrication

PDMS microdevices were fabricated following our protocol described in [34]. SU-8 photolithography was used to fabricate both the top and bottom layers of the microfluidic channels. The PDMS was mixed following a ratio of 10:1 polymer:curing agent (Ellsworth Adhesives, Cat: 001003579103, Lot: H047J8Q025) and was subsequently poured on top of the master wafers containing the device architecture, which were then baked at 80 °C for 4 h. The PDMS was then removed from the wafers following polymerization and the devices were assembled. The assembled devices were then bonded to a 50-mm glass bottom Petri dish (MatTek Corporation, P50G-1.5–30-F) using plasma bonding. Prior to use in cell culture the devices were sterilized with a 15-min exposure to ultraviolet light. 

### 4.3. Cell Culture and Maintenance

Commercial astrocytes were purchased and, to supplement the manufacturer’s GFAP-positive validation, we performed our own GFAP staining to further confirm that these cells were indeed astrocytes. Astrocytes were used between passages 0–5 and trypsinized at 90% confluence prior to experimentation. 

### 4.4. Astrocyte Culture within the Microdevice

Rat astrocytes were cultured in rat astrocyte basal medium containing growth serum (Cell Applications, Cat: R820-485, Lot: 160). To prepare experimental devices, a 0.05% trypsin solution (Gibco, Cat: 25300-062, Lot:2323053) was used to detach the cells. The cells were then resuspended within the rat astrocyte growth medium at the appropriate density. Next, a collagen gel which contained the rat astrocyte cells was prepared. The collagen gel contained 40 uL of 10× phosphate-buffered saline (PBS), 8 uL of 1 M NaOH, 300 uL of collagen type I (Corning Collagen Type I Rat Tail High Concentration 8.43 mg/mL, 354249), 52 uL of deionized (DI) water, and 400 uL of cell suspension. The collagen gel mixture was injected into the microfluidic device chambers and left at room temperature for 15 min to polymerize. Rat astrocyte growth medium (200 uL for PDMS devices and 8 mL for Polystyrene devices) was added to the cell culture dish (Corning, Ref: 353003, Lot: 6318018) and placed in an incubator at 37 °C with 5% CO_2_. We did not manipulate the oxygen content in the media. However, the media could be exposed to a gas mixture containing a defined percentage of oxygen to control the percentage of oxygen perfused through the system, which in turn, would allow the user to test the effect of multiple oxygen concentrations

### 4.5. Cell Viability

Calcein acetoxymethyl ester (Thermo Fisher Scientific, Ref: C3100 MP, Lot: 2286887) and propidium iodide (Thermo Fisher Scientific, P1304 MP) were used to measure cell viability and were prepared following the instructions provided by the supplier. The working solutions of CAM and PI were prepared at 1:1000 and 1:500, respectively, by dissolving the stock solutions in PBS. The microdevices were disassembled to expose the collagen gel. The solution of CAM/PI was added homogeneously on top of the collagen gel and allowed to rest for 15 min prior to imaging. Cell viability was measured using a Nikon TiE microscope with the temperature set to 37 °C and CO_2_ control set to 5%.

### 4.6. Calcium Imaging

To measure astrocyte signaling capacity, in accordance with manufacturer instructions, a working solution of fluo-4 (Fisher Scientific, Ref: F14201, Lot: 2335579) was diluted 1:500 in rat astrocyte basal medium containing growth serum (Cell Applications, Cat: R820-485, Lot: 160). The solution was perfused on top of the cell culture to completely cover the plate of cells and was left for a period of 45 min. The solution of fluo-4 and media was then removed and rat astrocyte media containing growth serum was perfused again prior to imaging. The cell culture was imaged using a Nikon TiE microscope, with the system set to 5% CO_2_ and 37 °C. Calcium spikes were analyzed by normalizing their fluorescence intensity using time-lapse microscopy (images every 5 s for 10 min). To detect pulses in calcium signaling, fluorescence intensity was normalized with respect to the cell’s fluorescence baseline. In order to be considered as calcium pulse, changes in calcium-induced fluorescence intensity had to be larger than a 10% over their corresponding baseline. This approach ensured all cells were considered in the analysis regardless of their baseline intensity.

### 4.7. Morphological Analysis

Image J software was utilized to perform morphological analysis. The “Area” and “Area Fraction” functions were used to determine the area of the astrocytes in experimental images. Then, the background of the images was subtracted using the “Subtract Background” operation. Finally, the “Apply Threshold” function was manually adjusted, and the “Analyze Particles (Circularity)” function was used to determine the circularity of astrocytes.

### 4.8. Hypoxia Staining 

To measure gradients of oxygen, a collagen gel was prepared containing rat astrocytes at a density calculated to be 5 Million cells/mL. In accordance with manufacturer instructions, a working solution of hypoxia reagent (Thermo Fisher Scientific, Ref: I14834, Lot: 2403679), was diluted 1:500 and added to the collagen gel and rat astrocyte basal medium containing growth serum (Cell Applications, Cat: R820-485, Lot: 160). The devices were imaged at time equal to zero and at time 24 h after the hypoxia stain. 

### 4.9. Statistical Analysis 

All experiments contained at least three biological replicates. The results are presented to show the mean +/− standard deviation. The analysis on the data was performed using Excel and the threshold for statistical significance was set at *p* < 0.05. The normal distribution assumption for statistical tests was confirmed by the Kolmogorov–Smirnov test. For multiple nonparametric comparisons, a Kruskal–Wallis test was performed followed by the Mann–Whitney U test, and for parametric comparisons one-way ANOVA test was used. T-test was used for paired parametric comparisons.

## Data Availability

All the data described in this manuscript is publicly available in the manuscript figures and supplementary materials. Data will also be made available upon request to the corresponding author.

## Supplementary Materials

The following supporting information can be downloaded at: https://www.mdpi.com/article/10.3390/cells11152356/s1. Figure S1 shows astrocytes stained with an anti-GFAP antibody, which demonstrates their astrocytic origin. Figure S2 shows the hypoxia analysis in the PDMS and the polystyrene-based microdevices. PDMS oxygen permeability led to no oxygen gradient formation across the microchamber, whereas in the polystyrene-based microdevices we observed the formation of an oxygen gradient after 24 hours in culture. Figure S3 shows that the number of astrocytes in the 3D hydrogel did not increase over a period of 3 days in culture. Figure S4 illustrates the variety of morphologies exhibited by astrocytes recovered from the microdevice.
