# Supplementary material for: Microfluidic Model to Evaluate Astrocyte Activation in Penumbral Region following Ischemic Stroke
Source: Cells. 2022 Jul 31;11(15):2356. doi: 10.3390/cells11152356 (PMC9367413; doi:10.3390/cells11152356)
Supplement: Supplementary file 1 [file cells-11-02356-s001.zip › cells-1817863-supplementary.pdf]

cells

Article

Microfluidic Model to Evaluate Astrocyte Activation in Penumbral Region following Ischemic Stroke

Kathryn M. Denecke [1,2], Catherine A. McBain [3], Brock G. Hermes [1], Sireesh Kumar Teertam [2], Mehtab Farooqui [1], María Virumbrales-Muñoz [1], Jennifer Panackal [2], David J. Beebe [1,4], Bolanle Famakin [2,†] and Jose M. Ayuso [3,4,*,†]

1 Department of Pathology and Laboratory Medicine, University of Wisconsin-Madison, Madison, WI 53705, USA; kdenecke@wisc.edu (K.M.D.); bhermes@wisc.edu (B.G.H.); mfarooqui@wisc.edu (M.F.); virumbralesm@wisc.edu (M.V.-M.); djbeebe@wisc.edu (D.J.B.)
2 Department of Neurology, University of Wisconsin-Madison, Madison, WI 53705, USA; teertam@neurology.wisc.edu (S.K.T.); panackal@wisc.edu (J.P.); famakin@neurology.wisc.edu (B.F.)
3 Department of Dermatology, University of Wisconsin-Madison, Madison, WI 53705, USA; cmcbain@dermatology.wisc.edu
4 UW Carbone Cancer Center, University of Wisconsin-Madison, Madison, WI 53705, USA
* Correspondence: ayusodomingu@wisc.edu
† These authors contributed equally to this work.

Supplementary Materials: Figure 1A shows astrocytes stained with an anti-GFAP antibody, which demonstrates their astrocytic origin. Figure 2A shows the hypoxia analysis in the PDMS and the polystyrene-based microdevices. PDMS oxygen permeability led to no oxygen gradient formation across the microchamber, whereas in the polystyrene-based microdevices we observed the formation of an oxygen gradient after 24 hours in culture. Figure A3 shows that the number of astrocytes in the 3D hydrogel did not increase over a period of 3 days in culture. Figure A4 illustrates the variety of morphologies exhibited by astrocytes recovered form the microdevice.

check for updates

Citation: Denecke, K.M.; McBain, C.A.; Hermes, B.G.; Teertam, S.K.; Farooqui, M.; Virumbrales-Muñoz, M.; Panackal, J.; Beebe, D.J.; Famakin, B.; Ayuso, J.M. Microfluidic Model to Evaluate Astrocyte Activation in Penumbral Region following Ischemic Stroke. *Cells* **2022**, *11*, 2356. https://doi.org/10.3390/cells11152356

Academic Editors: Sooyeon Lee and Kevin Leclerc

Received: 29 June 2022
Accepted: 28 July 2022
Published: 31 July 2022

Publisher's Note: MDPI stays neutral with regard to jurisdictional claims in published maps and institutional affiliations.

Copyright: © 2022 by the authors. Licensee MDPI, Basel, Switzerland. This article is an open access article distributed under the terms and conditions of the Creative Commons Attribution (CC BY) license (https://creativecommons.org/licenses/by/4.0/).

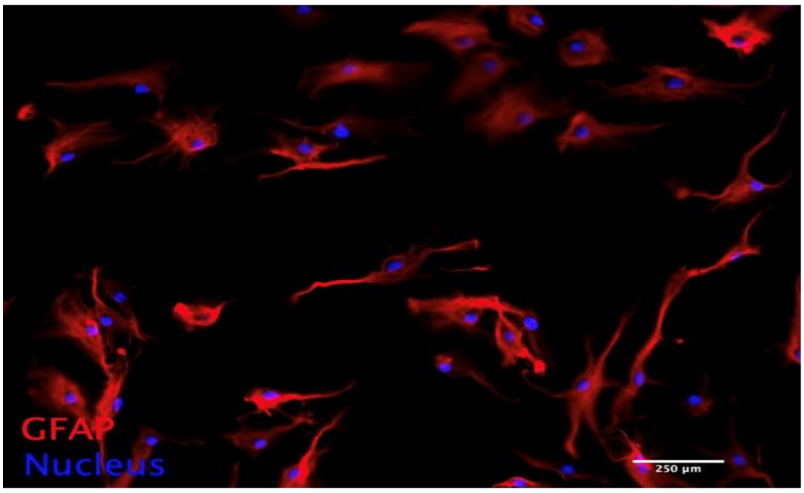

Figure S1. Primary rat cortical astrocytes cultured on Poly-L-lysine coated coverslips were stained with anti-GFAP to confirm astrocyte origin. The GFAP stain is shown in red and DAPI stained nu-clei are shown in blue.

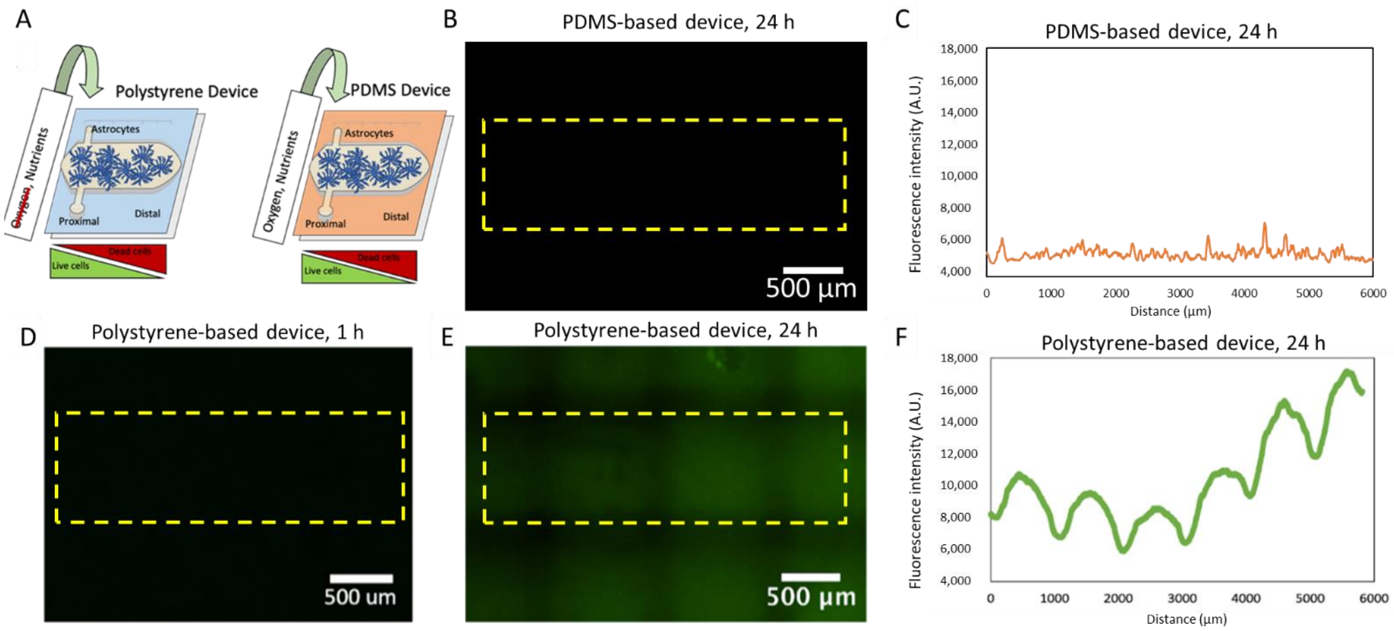

Figure S2. (**A**) Schematic of the polystyrene and PDMS-based microdevices. (**B**,**C**) Hypoxia-sensing die within the PDMS-based microdevice imaged after 24 h in culture. Graph shows the hypoxia-induced fluorescence across the delimited region (yellow rectangle) in (**B**,**D**–**F**) Hypoxia-sensing die in the Polystyrene-based microdevice imaged at 1 and 24 h in culture. Graph shows the hypoxia-induced fluorescence across the delimited region (yellow rectangle) in E.

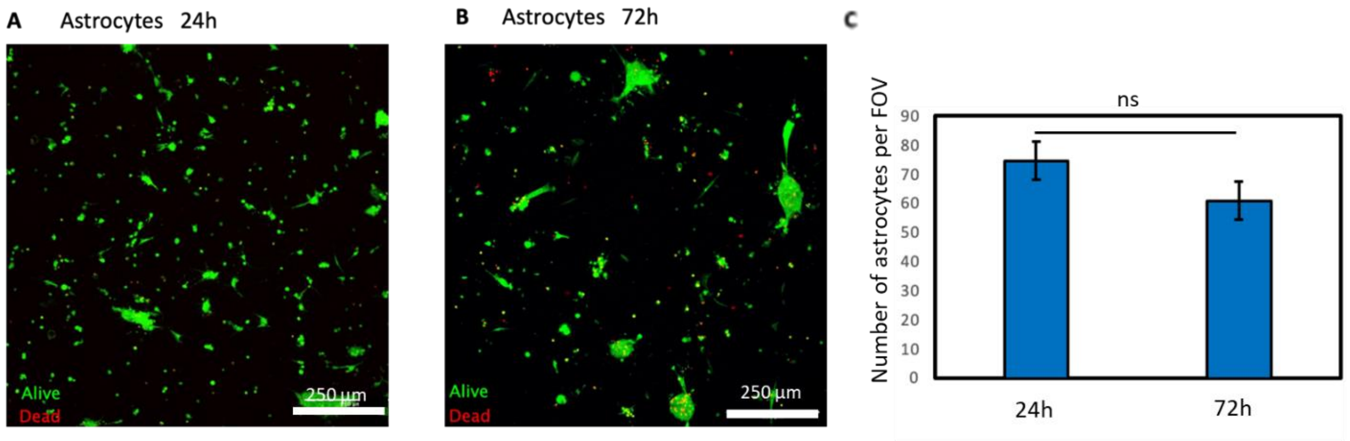

Figure S3. Astrocytes proliferation in the microdevice. (**A**,**B**) Confocal microscopy images show viabile and dead astrocytes in green and red respectivly after 24 and 72 h in culture. (**C**) Graph shows the number of astrocytes in the microdevice after 24 and 72 h in culture.

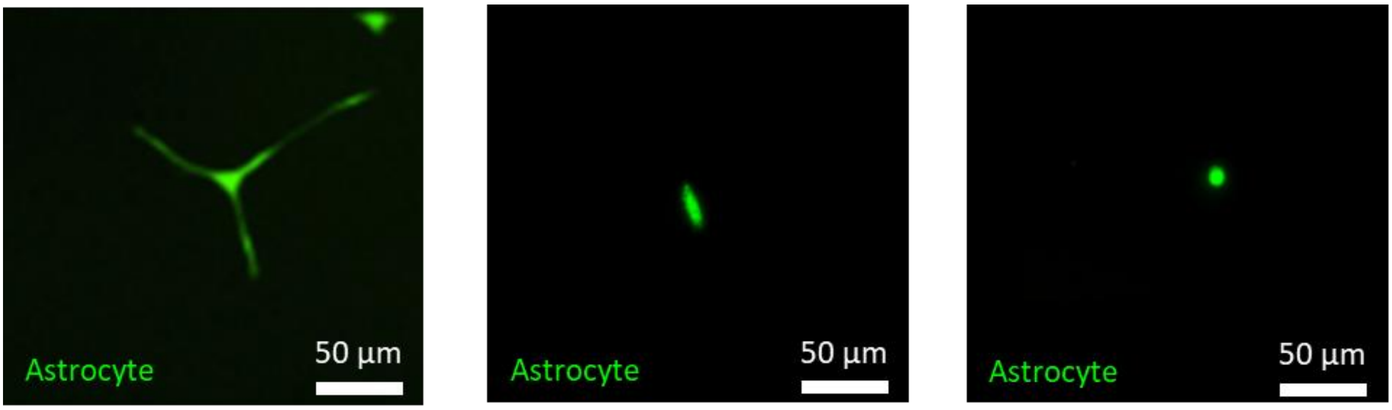

Figure S4. Astrocyte morphologies. Astrocytes were cultured in the microdevice for 3 days and then retrieved from the hydrogel and cultured in regular flasks for 5 days. Recovered astrocytes exhibited a broad range of cell morphologies.

References:

1. GBD 2015 Neurological Disorders Collaborator Group. Global, regional, and national burden of neurological disorders during 1990–2015: A systematic analysis for the Global Burden of Disease Study 2015. Lancet Neurol. 2017, 16, 877–897. https://doi.org/10.1016/S1474-4422(17)30299-5.
2. Katan, M.; Luft, A. Global Burden of Stroke. Semin. Neurol. 2018, 38, 208–211. https://doi.org/10.1055/s-0038-1649503.
3. Sacco, R.L.; Kasner, S.E.; Broderick, J.P.; Caplan, L.R.; Connors, J.J.; Culebras, A.; Elkind, M.S.; George, M.G.; Hamdan, A.D.; Higashida, R.T.; et al. An Updated Definition of Stroke for the 21st Century: A statement for healthcare professionals from the American heart association/American stroke association. Stroke 2013, 44, 2064–2089. https://doi.org/10.1161/str.0b013e318296aeca.
4. Revuelta, M.; Elicegui, A.; Moreno-Cugnon, L.; Bührer, C.; Matheu, A.; Schmitz, T. Ischemic stroke in neonatal and adult astrocytes. Mech. Ageing Dev. 2019, 183, 111147. https://doi.org/10.1016/j.mad.2019.111147.
5. Bhaskar, S.; Stanwell, P.; Cordato, D.; Attia, J.; Levi, C. Reperfusion therapy in acute ischemic stroke: Dawn of a new era? BMC Neurol. 2018, 18, 8. https://doi.org/10.1186/s12883-017-1007-y.
6. Donnan, G.A.; Fisher, M.; Macleod, M.; Davis, S.M. Stroke. Lancet 2008, 371, 1612–1623. https://doi.org/10.1016/s0140-6736(08)60694-7.
7. Gattringer, T.; Posekany, A.; Niederkorn, K.; Knoflach, M.; Poltrum, B.; Mutzenbach, S.; Haring, H.-P.; Ferrari, J.; Lang, W.; Willeit, J.; et al. Predicting Early Mortality of Acute Ischemic Stroke. Stroke 2019, 50, 349–356. https://doi.org/10.1161/strokeaha.118.022863.
8. Yousufuddin, M.; Young, N. Aging and ischemic stroke. Aging 2019, 11, 2542–2544. https://doi.org/10.18632/aging.101931.
9. Ovbiagele, B.; Goldstein, L.B.; Higashida, R.T.; Howard, V.J.; Johnston, S.C.; Khavjou, O.A.; Lackland, D.T.; Lichtman, J.H.; Mohl, S.; Sacco, R.L.; et al. Forecasting the Future of Stroke in the United States: A policy statement from the American Heart Association and American Stroke Association. Stroke 2013, 44, 2361–2375. https://doi.org/10.1161/str.0b013e31829734f2.
10. Sharrief, A.; Grotta, J.C. Stroke in the elderly. In Handbook of Clinical Neurology; Elsevier: Amsterdam, The Netherlands, 2019; Volume 167, pp. 393–418. https://doi.org/10.1016/b978-0-12-804766-8.00021-2.
11. Chamorro, Á.; Lo, E.H.; Renú, A.; van Leyden, K.; Lyden, P.D. The future of neuroprotection in stroke. J. Neurol. Neurosurg. Psychiatry 2021, 92, 129–135. https://doi.org/10.1136/jnnp-2020-324283.
12. Siracusa, R.; Fusco, R.; Cuzzocrea, S. Astrocytes: Role and Functions in Brain Pathologies. Front. Pharmacol. 2019, 10, 1114. https://doi.org/10.3389/fphar.2019.01114.
13. Becerra-Calixto, A.; Cardona-Gómez, G.P. The Role of Astrocytes in Neuroprotection after Brain Stroke: Potential in Cell Therapy. Front. Mol. Neurosci. 2017, 10, 88. https://doi.org/10.3389/fnmol.2017.00088.
14. Liddelow, S.A.; Guttenplan, K.A.; Clarke, L.E.; Bennett, F.C.; Bohlen, C.J.; Schirmer, L.; Bennett, M.L.; Münch, A.E.; Chung, W.-S.; Peterson, T.C.; et al. Neurotoxic reactive astrocytes are induced by activated microglia. Nature 2017, 541, 481–487. https://doi.org/10.1038/nature21029

15. Patabendige, A.; Singh, A.; Jenkins, S.; Sen, J.; Chen, R. Astrocyte Activation in Neurovascular Damage and Repair Following Ischaemic Stroke. Int. J. Mol. Sci. 2021, 22, 4280. https://doi.org/10.3390/ijms22084280.
16. Guo, H.; Fan, Z.; Wang, S.; Ma, L.; Wang, J.; Yu, D.; Zhang, Z.; Wu, L.; Peng, Z.; Liu, W.; et al. Astrocytic A1/A2 paradigm participates in glycogen mobilization mediated neuroprotection on reperfusion injury after ischemic stroke. J. Neuroinflammation 2021, 18, 230. https://doi.org/10.1186/s12974-021-02284-y.
17. Ayuso, J.M.; Park, K.-Y.; Virumbrales-Muñoz, M.; Beebe, D.J. Toward improved in vitro models of human cancer. APL Bioeng. 2021, 5, 010902. https://doi.org/10.1063/5.0026857.
18. Taha, A.; Bobi, J.; Dammers, R.; Dijkhuizen, R.M.; Dreyer, A.Y.; van Es, A.C.; Ferrara, F.; Gounis, M.J.; Nitzsche, B.; Platt, S.; et al. Comparison of Large Animal Models for Acute Ischemic Stroke: Which Model to Use? Stroke 2022, 53, 1411–1422. https://doi.org/10.1161/strokeaha.121.036050.
19. Ayuso, J.M.; Virumbrales-Munoz, M.; McMinn, P.H.; Rehman, S.; Gomez, I.; Karim, M.R.; Trusttchel, R.; Wisinski, K.B.; Beebe, D.J.; Skala, M.C. Tumor-on-a-chip: A microfluidic model to study cell response to environmental gradients. Lab Chip 2019, 19, 3461–3471. https://doi.org/10.1039/c9lc00270g.
20. Zirath, H.; Rothbauer, M.; Spitz, S.; Bachmann, B.; Jordan, C.; Müller, B.; Ehgartner, J.; Priglinger, E.; Mühleder, S.; Redl, H.; et al. Every Breath You Take: Non-invasive Real-Time Oxygen Biosensing in Two- and Three-Dimensional Microfluidic Cell Models. Front. Physiol. 2018, 9, 815. https://doi.org/10.3389/fphys.2018.00815.
21. Liu, Z.; Chopp, M. Astrocytes, therapeutic targets for neuroprotection and neurorestoration in ischemic stroke. Prog. Neurobiol. 2016, 144, 103–120. https://doi.org/10.1016/j.pneurobio.2015.09.008.
22. Mergenthaler, P.; Lindauer, U.; Dienel, G.A.; Meisel, A. Sugar for the brain: The role of glucose in physiological and pathological brain function. Trends Neurosci. 2013, 36, 587–597. https://doi.org/10.1016/j.tins.2013.07.001.
23. Ding, S. Ca2+ Signaling in Astrocytes and its Role in Ischemic Stroke. In Glutamate and ATP at the Interface of Metabolism and Signaling in the Brain; Advances in Neurobiology; Springer: Cham, Switzerland, 2014; Volume 11, pp. 189–211. https://doi.org/10.1007/978-3-319-08894-5_10.
24. Stanzione, R.; Cotugno, M.; Bianchi, F.; Marchitti, S.; Forte, M.; Volpe, M.; Rubattu, S. Pathogenesis of Ischemic Stroke: Role of Epigenetic Mechanisms. Genes 2020, 11, 89. https://doi.org/10.3390/genes11010089.
25. Zhao, L.-R.; Willing, A. Enhancing endogenous capacity to repair a stroke-damaged brain: An evolving field for stroke research. Prog. Neurobiol. 2018, 163–164, 5–26. https://doi.org/10.1016/j.pneurobio.2018.01.004.
26. Morrison, H.W.; Filosa, J.A. Stroke and the neurovascular unit: Glial cells, sex differences, and hypertension. Am. J. Physiol. Cell Physiol. 2019, 316, C325–C339. https://doi.org/10.1152/ajpcell.00333.2018.
27. Zhao, Z.; Nelson, A.R.; Betsholtz, C.; Zlokovic, B.V. Establishment and Dysfunction of the Blood-Brain Barrier. Cell 2015, 163, 1064–1078. https://doi.org/10.1016/j.cell.2015.10.067.
28. Virumbrales-Muñoz, M.; Ayuso, J.M.; Gong, M.M.; Humayun, M.; Livingston, M.K.; Lugo-Cintrón, K.M.; McMinn, P.; Álvarez-García, Y.R.; Beebe, D.J. Microfluidic lumen-based systems for advancing tubular organ modeling. Chem. Soc. Rev. 2020, 49, 6402–6442. https://doi.org/10.1039/d0cs00705f.
29. Oberheim, N.A.; Takano, T.; Han, X.; He, W.; Lin, J.H.C.; Wang, F.; Xu, Q.; Wyatt, J.D.; Pilcher, W.; Ojemann, J.; et al. Uniquely Hominid Features of Adult Human Astrocytes. J. Neurosci. 2009, 29, 3276–3287. https://doi.org/10.1523/jneurosci.4707-08.2009.
30. Ayuso, J.; Rehman, S.; Farooqui, M.; Virumbrales-Muñoz, M.; Setaluri, V.; Skala, M.; Beebe, D. Microfluidic Tumor-on-a-Chip Model to Study Tumor Metabolic Vulnerability. Int. J. Mol. Sci. 2020, 21, 9075. https://doi.org/10.3390/ijms21239075.
